# Supplementary material for: Tailoring elastic and inelastic collisions of relativistic antiferromagnetic domain walls
Source: Sci Rep. 2023 Nov 30;13:21153. doi: 10.1038/s41598-023-47662-z (PMC10689819; doi:10.1038/s41598-023-47662-z)
Supplement: Supplementary file 1 — Supplementary Information. [file 41598_2023_47662_MOESM1_ESM.pdf]

Tailoring Elastic and Inelastic Collisions of Relativistic Antiferromagnetic Domain Walls

Rubén M. Otxoa, Gen Tatara, Pierre E. Roy & Oksana Chubykalo-Fesenko

Energy consumption per computation

The efficiency of a computing event is typically given in terms of the quality factor, Q, defined here as the ratio between the energy carried by the AF DWs and the energy dissipated in a computation event. In principle, as the damping is compensated by the action of the SO-field with barely any spin wave emission[1] at the moment of the collision event, it is expected the Q-factor to be close to one, as obtained in current CMOS technology. For conventional computers large fraction of the energy associated with certain computation is drawn from the power supply and is dissipated into environment. This causes a poor waste-heat management and acts in detriment in the processing speed. Therefore, the ideal case scenario would correspond to a minimal energy dissipated per computation and the use of spintronics-based AF DW dynamics seems to meet this condition.

In this supplementary note we aim to calculate the power and energy consumed per computation event based on the resistivity of the material, injected current density and logic operation performed by an elastic collision event among the relativistic atiferromagnetic domain walls (DWs).

The values used for the resistivity, ρ, have been reported experimentally for Mn_2Au in [2] as a function of the level of disorder by the excess of Au atoms. The resistance is given by

$$R = \rho \frac{l}{S}, \tag{1}$$

where ρ is the resistivity, l is the length of the computational cell and S is its section. We take l= 200 nm and S=50 nm^2. In our case, we consider a cell that is able to contain two DWs and it is sufficiently large so that the DWs are able after the initial inertial regime to achieve steady speeds which are larger than the critical speed to observe elastic collision among them. For a disorder below 1% due to Au atoms excess [2], $\rho \approx 1\mu\Omega$cm. In such a case the resistance is R= 4Ω.

On the other hand the critical current flowing by the track is

$$I_{\rm crit} = j_{\rm crit.}.S, \tag{2}$$

where $j_{\rm crit}$ is the critical current density above which the DWs can elastically collide. From our full atomistic spin dynamics simulations, confirmed by analytical calculations, the minimum SO-field ($H_{\rm crit}$) for a tunnel process to occur is 22 mT. As 2 mT of SO-field value ($H_{\rm so}$) corresponds to a current density, $j \approx 10^{11}$A/m^2 [3] then $j_{\rm crit} \approx 10^{12}$A/m^2. (Note that the current pulse would have 1-10 ps duration and thus no large Joule heating is expected.) Therefore, the total critical current is $I_{\rm crit}$=500 mA. From this value we easily calculate the power as

$$P = RI^2, \tag{3}$$

where by substitution one finds, P=1μW. The energy estimated per computation can be obtained by accounting for the time, $t_{\rm c}$ that the DWs need to propagate to the collision point. The energy will be simply the product between the power and $t_{\rm c}$ as follows

$$E_{\rm comp.} = Pt_{\rm c}. \tag{4}$$

Furthermore, the time $t_{\rm c}$ can be estimated as the time taken by the two DWs to travel the distance 100 nm each to the collision point. Assuming a travelling steady speed equal to the critical velocity, $v_{\rm crit.}$=35

km/s = 35 nm/ps, each DW takes about 3 ps before the collision occurs. Subsequently, the energy per computation event is: $E_{\text{comp.}}$=1μW·3ps = 2*10^{-18}J = 3 aJ.

We can compare the collision-based relativistic soliton computing with other technological approaches. For neuroinspired computing based on spin-torque nano-oscillators, from Romera *el al.* [4], one can extract the energy per oscillation/operation to be hundreds of aJ which is 2 orders of magnitude larger than the one obtained from our proposal. Furthermore Raab *et al.* [5] use topologically protected magnetic textures to carry out computation where, the energy per operation is circa 50 aJ, which is an order of magnitude larger than in our case. Note that the energetic efficiency for antiferromagnetic domain walls comes in the first place from their large velocities, resulting in small computational time t_c. Therefore, it seems that than intrinsic relativistic nature of antiferromagnetic DWs in conjunction with topological features not only show formal solitonic behaviour (elastic collision) but also potential for computational approaches that outperform current ferromagnetic-based platforms.

Supplementary References

[1] R. M. Otxoa, R. Rama-Eiroa, P. Roy, G. Tatara, O. Chubykalo-Fesenko, and U. Atxitia, Physical Review Research **3**, 043069 (2021).

[2] S. Y. Bodnar, L. Šmejkal, I. Turek, T. Jungwirth, O. Gomonay, J. Sinova, A. Sapozhnik, H.-J. Elmers, M. Kläui, and M. Jourdan, Nature communications **9**, 348 (2018).

[3] J. Železnỳ, H. Gao, K. Vỳbornỳ, J. Zemen, J. Mašek, A. Manchon, J. Wunderlich, J. Sinova, and T. Jungwirth, Physical review letters **113**, 157201 (2014).

[4] M. Romera, P. Talatchian, S. Tsunegi, F. Abreu Araujo, V. Cros, P. Bortolotti, J. Trastoy, K. Yakushiji, A. Fukushima, H. Kubota, *et al.*, Nature **563**, 230 (2018).

[5] K. Raab, M. A. Brems, G. Beneke, T. Dohi, J. Rothörl, F. Kammerbauer, J. H. Mentink, and M. Kläui, Nature Communications **13**, 6982 (2022).
